# In Situ Alkali Metal Exfoliation‐Coupled Dilute CO_2_ Electrolysis to Synthesize Interlayer‐Expanded Graphite for High‐Rate Lithium Storage

**DOI:** 10.1002/advs.76734

**Published:** 2026-07-23

**Authors:** Hao Zha, Xiaodan Zhang, Xinyu Li, Jiajun Li, Yuxin Wu, Xiaoyang Wang, Huayi Yin, Dihua Wang, Bowen Deng

**Affiliations:** ^1^ School of Resource and Environmental Sciences Wuhan University Wuhan P. R. China; ^2^ Hubei International Scientific and Technological Cooperation Base of Sustainable Resource and Energy Wuhan University Wuhan P. R. China

**Keywords:** CO_2_ electroreduction, graphite interlayer, high rate performance, LIBs, molten salt

## Abstract

Converting CO_2_ into ideal carbon anode materials for fast lithium storage brings a win‐win prospect toward energy and environment domains. Here, we propose a one‐pot stepwise strategy to synthesize expanded *d*‐spacing (up to 0.405 nm) graphite by direct electroreduction of dilute CO_2_ (e.g., 10 vol%, close to practical industrial flue gas) in molten electrolytes, where the as‐formed CO_2_‐derived graphite with pristine interlayer spacing was enlarged by the sequential in situ exfoliation of alkali metals. Owing to the predominant thermodynamic tendency, dilute CO_2_ was preferentially electro‐transformed into carbon materials at a promising current density of 100 mA/cm^2^. Serving as a LIBs anode, the obtained graphite with expanded *d*‐spacing exhibited a relatively high lithium storage capacity (152 mAh/g) at a high charge/discharge current density of 2000 mA/g, fivefold compared with that of commercial graphite, reaching a retention rate of 91% after 500 cycles. This work provides insights on rational design of high‐rate lithium storage anode for next‐generation LIBs industry with a lower carbon footprint.

## Graphical abstract

Converting CO_2_ into advanced carbon materials capable of meeting the rapid lithium storage demands of lithium‐ion batteries creates a mutually beneficial scenario for both energy and environmental sectors. Here, we propose a stepwise CO_2_ electrolysis approach to transform dilute CO_2_ into interlayer‐expanded graphite using molten salt electrolyzers, demonstrating excellent rate performance and cycling stability under high‐rate conditions.

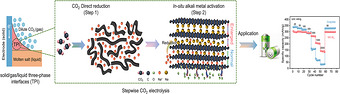

## 1 Introduction

With the growing concerns over climate change and energy security, efficient energy storage technologies, particularly rechargeable batteries, have garnered widespread attention and exhibit significant potential in market prospects [1], of which rapid expansion is largely attributed to the successful development of lithium‐ion batteries (LIBs) due to their high energy efficiency and long cycle life [2, 3]. However, the current long charging duration of LIBs poses challenges in meeting the demand for high spatiotemporal efficiency in large‐scale energy storage applications, such as electric vehicles and power grids [4, 5]. Although optimizing the charging process can significantly reduce charging time, further shortening the charging duration becomes difficult due to lithium deposition on graphite anodes [6]. This phenomenon typically occurs when the charging rate increases, leading not only to severe capacity degradation but also to an increased risk of short circuits [7, 8]. Additionally, metallic lithium reacts with the electrolyte, generating a substantial amount of solid electrolyte interphase, which in turn leads to the continuous consumption of active lithium and a rapid decline in the lithium storage capacity [9, 10]. This is primarily attributed to the slow kinetics of Li^+^ intercalation into graphite, mainly due to the relatively narrow interlayer spacing of graphite (approximately 0.334 nm) [11, 12].

The microstructure engineering of graphite materials has paved a significant solution strategy in the development of fast‐charging batteries to resolve the above‐mentioned issue [13]. In particular, dual graphite batteries that utilize graphite derivatives as anode and cathode active materials have emerged as representatives of high‐power battery technology [14]. These batteries achieve energy storage through the intercalation/deintercalation of cations and anions during charge and discharge processes [15]. However, such battery systems still face challenges due to the sluggish ionic kinetics caused by the narrow interlayer spacing of graphite materials. Interlayer engineering, defined as the regulation of the interlayer spacing of graphite materials, is regarded as an effective approach to enhance the electrochemical kinetic performance (such as rate capability) by optimizing the diffusion pathways in the graphite anode [16]. Currently, the adjustment of the interlayer spacing of graphite can be achieved through physical expansion methods, which involve the insertion of organic/inorganic molecules (such as ammonium ions, ionic liquids, etc.) into the interlayers [17, 18]. Although these methods can effectively regulate the interlayer spacing, the carbon footprint during the preparation process for commercial LIB carbon anode materials remains a relatively high level, thus failing to provide a reliable and comprehensive solution for the development of fast‐charging LIB anode materials.

The conversion of greenhouse gas CO_2_ into advanced carbon materials that can meet the rapid lithium storage demands of LIBs not only promises to alleviate the shortage of ideal carbon anode materials but also significantly reduces the carbon footprint of the LIBs industry, achieving a win‐win prospect. Renewable energy‐driven CO_2_ electroreduction technology (CO_2_RR) is one of the most effective method for converting CO_2_ into high‐value‐added products and establishing an artificial carbon cycling. To be specific, in room‐temperature aqueous CO_2_RR systems, CO_2_ is primarily converted into carbon‐containing liquid fuels through hydrogenation [19], whereas in high‐temperature solid oxide electrolytes, CO_2_ is converted into gaseous products such as CO [20]. Among various electrolyte systems, high‐temperature molten salts are the only electrolysis system capable of continuously converting CO_2_ into advanced carbon materials [21]. This unique electrolyte system always operates via an indirect conversion mechanism, in which captured CO_2_ (in the form of carbonate ions) is electrochemically reduced to carbon products at the cathode. This process typically utilizes reactions carried out in carbonate‐containing melts co‐existed with lithium (Li^+^) or calcium (Ca^2+^) ions [22]. With the assistance of transition metal nucleation, heterogeneous heteroatom doping, and Joule heating during the electrolysis, regular graphitic carbon products such as carbon nanotubes, graphite, and graphene can be achieved, respectively [23, 24, 25]. These CO_2_‐derived carbon materials hold great promise for applications in energy fields such as supercapacitors and lithium‐ion batteries [26]. For example, as an LIBs anode material, its lithium storage specific capacity can be competitive with that of commercial graphite, reaching a specific capacity exceeding 400 mAh/g at a charge‐discharge current density of 200 mA/g [8]. However, these CO_2_‐derived carbon materials still lack effective regulation methods to expand the interlayer spacing of graphite, making it difficult to meet the demands of rapid charge/discharge. On the other hand, industrial flue gas with a low CO_2_ content (10–20 vol%) accounts for 62% of annual global greenhouse gas emissions [27]. But the existing CO_2_RR technologies primarily utilize high‐content CO_2_ (usually > 90 vol%) to drive a promising reaction kinetics [28, 29]. This tedious process can increase the complexity and financial cost of the CO_2_RR technology. Therefore, achieving the direct high‐value utilization of dilute CO_2_, particularly its conversion into carbon materials capable of rapid lithium storage, remains a great challenge.

By fully leveraging the unique advantages of high‐temperature molten salt electrolysis systems in CO_2_ conversion to solid carbon, this work innovatively constructed a high‐temperature gas diffusion electrode that can directly electrochemically reduce dilute CO_2_ (content close to actual flue gas) into advanced carbon materials without the need for neither CO_2_ capture nor CO_2_ enrichment. By in situ employing a stepwise electrolysis method coupled with the alkali metal precipitation process, an exfoliation effect originating from either volume change or its shear effort was generated on the as‐formed graphite interlayers, achieving low‐cost, short‐process electrosynthesis of graphite with expanded lattice spacing features. This unique carbon material exhibited excellent rate performance and cycling stability under high‐rate charge and discharge conditions, capable of meeting the demands for rapid lithium storage. Furthermore, this work revealed the thermodynamic principles of direct electrochemical reduction of dilute CO_2_ towards carbon materials, and also analyzed the correlations between the graphitization of carbon materials and the enlargement of lattice spacing under different electrolysis conditions. The application performance of CO_2_‐derived carbon products with different lattice spacing characteristics as LIB anode materials were also investigated. This work not only deepens the fundamental understanding of the dilute CO_2_ utilization processes but also provides new insights for the next‐generation lithium‐ion battery anode materials with rapid charge/discharge features at a low carbon footprint level.

## 2 Results and Discussion

### 2.1 Thermodynamic Analysis and Electrochemical Measurements

**FIGURE 1:**
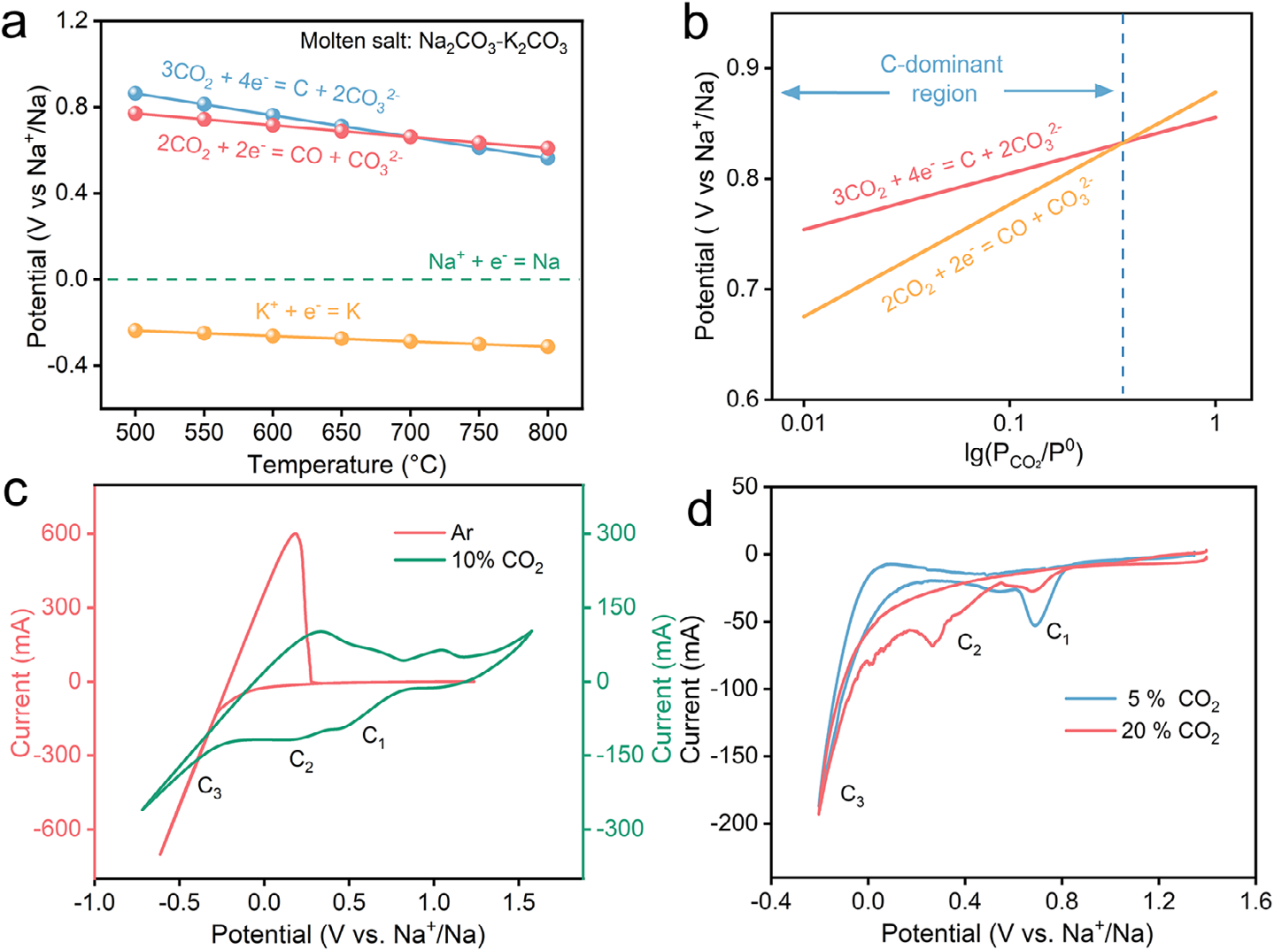
(a) Theoretical potentials for possible cathodic reactions in molten Na_2_CO_3_–K_2_CO_3_. (b) Correlations between CO_2_ partial pressure and theoretical potential. Cyclic voltammetry profiles under different atmospheres (c) and various CO_2_ contents (d) on a Ni foam working electrode. Scan rate: 50 mV/s.

To obtain the expanded *d*‐spacing graphite by a one‐pot stepwise electrolysis strategy, where CO_2_ will be electrochemically reduced at the first step and alkali metal evolution will be sequentially occurred, the electrolyte supporting the target reaction processes must be deliberately designed. To achieve these attempts, here a direct CO_2_ electrolysis method (Equation 1) was proposed preliminarily in molten Na_2_CO_3_‐K_2_CO_3_, not only because the electrochemical reaction sequence is suitable (see more preferential potential of CO_2_‐to‐carbon conversion in Figure 1a), but also because an undesirable side reaction originating from the indirect CO_2_ electrolysis, the electroreduction of the carbonate ions via Equation 3, can be avoided in a Li^+^/Ca^2+^‐free electrolyte (Figure S2), allowing alkali metal evolution a straightforward reaction (Equation 5) in the absence of CO_2_.

Direct CO_2_ electrolysis toward carbon:

(1) $$CO_{2}\left(g\right)=C+O_{2}\left(g\right)$$

(2) $$3CO_{2}\left(g\right)+4e^{-}=C+2{\mathrm{CO}}_{3}^{2-}$$

Indirect CO_2_ electrolysis:

(3) $$3Li_{2}CO_{3}=3Li_{2}O+C+2CO_{2}+O_{2}\left(g\right)$$

(4) $${\mathrm{CO}}_{3}^{2-}+4e^{-}=C+3O^{2-}$$

Alkali metal evolution:

(5)

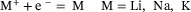




Noting that CO will also be probably obtained during CO_2_ electrolysis, a feasible countermeasure should be considered to steer the product selectivity toward carbon. Based on Nernst Equation (see details in the Supporting Information), the theoretical cathodic potential is dependent on CO_2_ partial pressure, as illustrated in Figure 1b, it is interesting to find that the CO_2_RR selectivity is highly associated with the applied CO_2_ partial pressure. Particularly, carbon will be a predominant product under a relatively low CO_2_ partial pressure, because the theoretical potential of CO_2_‐to‐carbon conversion (E_CO2/C_) is more favorable. By contrast, the potential of CO_2_‐to‐CO conversion (E_CO2/CO_) becomes more preferential under a high CO_2_ partial pressure. These observations indicate that regulating CO_2_ partial pressure can well steer the product selectivity. To be specific, a dilute CO_2_ will facilitate CO_2_‐to‐carbon conversion. To achieve the above‐mentioned attempts, we speculated that establishing a high‐temperature gas diffusion electrode (HT‐GDE) can enable a direct electroreduction of CO_2_, where alkali metal evolution can sequentially in situ take place by switching the atmosphere from CO_2_ to an Ar atmosphere, particularly in molten Na_2_CO_3_‐K_2_CO_3_.

A HT‐GDE enables the effective constructions of three‐phase interfaces (TPI), which are composed of gas (gaseous reactants)/liquid (electrolyte)/solid (electrode) phases, serving as the core reaction sites for electron transfer and mass exchange (Figure S3). Before applying a qualified HT‐GDE, we evaluated the electrochemical performance of TPI in molten Na_2_CO_3_‐K_2_CO_3_ by cyclic voltammetry (CV) under different atmospheres (Figure S3), where a Ni foam sheet was selected as a working electrode due to its excellent electronic conductivity and low cost. As shown in Figure 1c, it is evident that only a single cathodic peak C_3_ related to the alkali metal evolution was observed. However, the situation significantly changed when an atmosphere containing 10% CO_2_ was introduced. As can be clearly seen, there were two extra cathodic peaks C_1_ and C_2_, which should be related to CO_2_RR toward carbon and CO, respectively. The CVs under different CO_2_ contents in Figure 1d showed more evidences: the cathodic current of peak C_1_ (CO_2_ to carbon) was more evident under a relatively lower CO_2_ content while that of peak C_2_ (CO_2_ to CO) was decreased. These observations were consistent with the results of above‐mentioned thermodynamic analysis and our hypothesis, suggesting that a stepwise electrolysis, involving CO_2_RR (Step 1) and its sequential alkali metal evolution (Step 2), can be achieved simply by shifting the atmosphere from a low content CO_2_ to a CO_2_‐free one on TPI enabled by HT‐GDE.

### 2.2 Alkali Metal Exfoliation Coupled Stepwise CO_2_ Electrolysis

**FIGURE 2:**
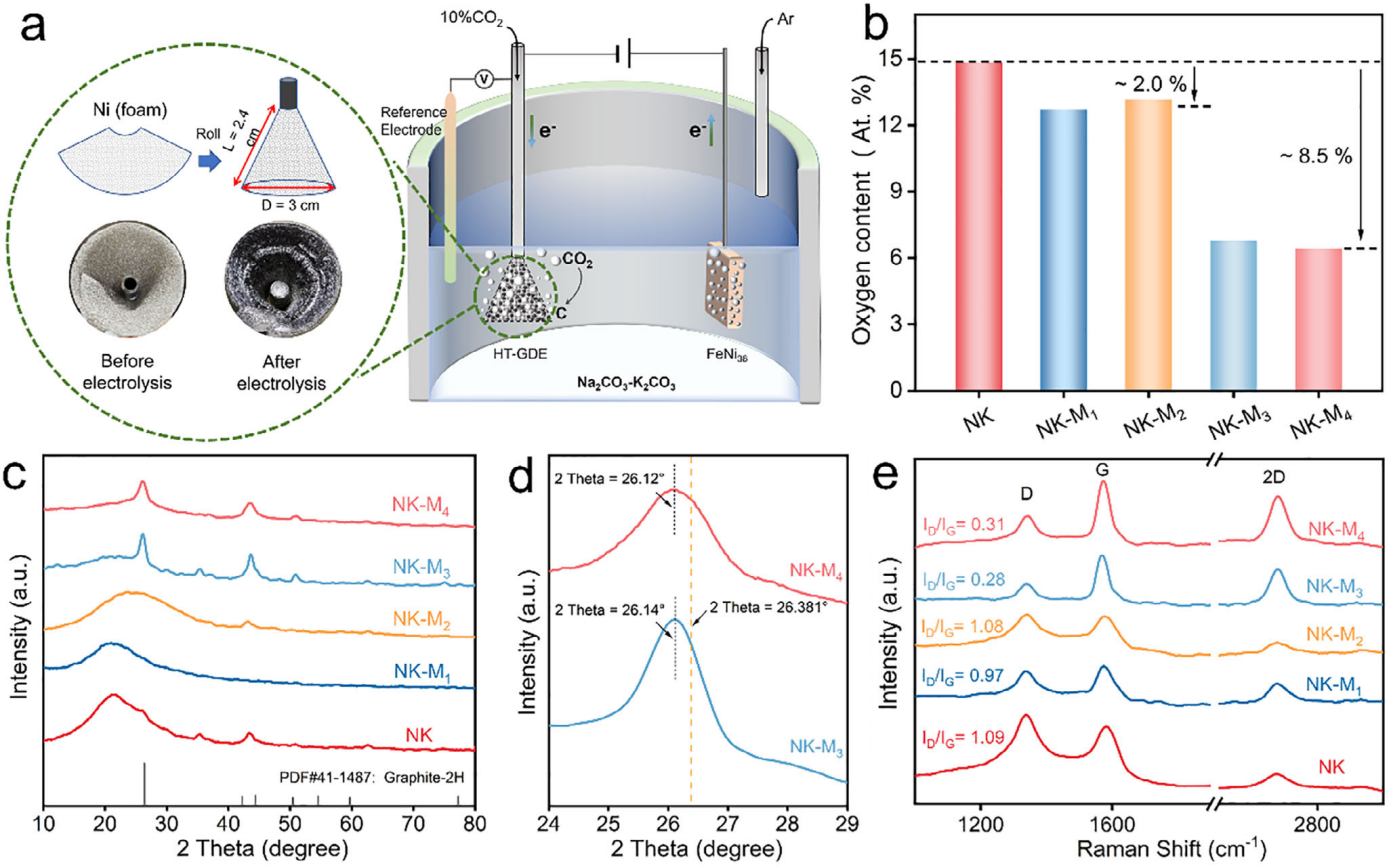
(a) Schematic illustration of stepwise CO_2_ electrolysis. (b) Oxygen content of different carbon products based on EDS analysis. XRD patterns (c, d) and Raman spectroscopy (e) of carbon products obtained under different conditions.

At Step 1, to achieve a continuous and stable CO_2_‐to‐carbon conversion, a well‐defined HT‐GDE was established by promoting the geometric configuration of Ni foam from a flat sheet‐like appearance to a three‐dimensional funnel‐shaped structure (Figure 2a), where 10% CO_2_ was consistently introduced by a hollow stainless steel tube centered over the top of funnel‐shaped Ni foam during CO_2_ electrolysis. The unique geometric design of the deliberately constructed cathode structure provides ample space for carbon deposition and effectively addresses the issue of pore blockage that may arise during this process. Generally, higher CO_2_ partial pressure, flow rate, elevated electrolysis temperature, and increased electrode specific surface area and porosity contribute positively to enhancing the upper limit of CO_2_RR current density. However, it is important to acknowledge a trade‐off effect between upper limit of current density and product selectivity. Specifically, excessively high CO_2_ partial pressure and electrolysis temperature may also lead to shift in the selectivity towards CO. Furthermore, the electrode materials must exhibit excellent high‐temperature corrosion resistance, and unobstructed pore channels must be maintained to ensure the stability of CO_2_ electrolysis. Besides, gas flow rate also impacts cathodic polarization. As illustrated in Figure S4, the cathodic potential gradually shifted in the positive direction as CO_2_ flow rate increased due to a chemical depolarization effect originating from CO_2_ capture by released oxide ions [30].

After that, electrolysis was carried out under an Ar atmosphere but without CO_2_ feeding to achieve alkali metal evolution at Step 2. The variations of cathodic potential and current density during stepwise electrolysis were recorded in real time. As shown in Figure S5, the cathodic potentials were more negative than 0 V (vs Na^+^/Na), suggesting that alkali metal evolution (e.g., sodium evolution) successfully occurred at Step 2. Moreover, the cathodic potential at Step 1 was always much more positive than that at Step 2, which was attributed to the different cathodic reactions (e.g., CO_2_RR and alkali metal evolution).

As can be found in Figure 2a, after the electrolysis at a current density of 100 mA/cm^2^ at step 1, black deposits can be observed on the surface of HT‐GDE. Energy‐dispersive X‐ray spectroscopy (EDS‐mapping) was employed to analyze the elemental composition and distribution of the obtained electrolytic products (Table S2). The results showed that the primary components of the materials with homogeneous distribution were C and O (Figure S6), collaboratively accounting for over 99 at.%, indicating that those black deposits were carbon materials. The oxygen content is an indicator for graphitization degree of carbon materials. According to EDS results (Figure 2b), it is interesting to find that the oxygen contents of the carbon (i.e., NK‐M*x* series samples) obtained after sequential alkali metal activation process were relatively lower compared with that of NK sample which was only a CO_2_RR product, suggesting an enhanced graphitization degree for the carbon products obtained by the stepwise CO_2_ electrolysis. This observation was attributed to a electro‐deoxidization effect during Step 2 [31]. X‐ray diffraction (XRD) patterns further confirmed the observation because plane (002) diffraction peaks located at ≈26° (Figure 2c), which were related to typical graphite feature, were found for the samples particularly obtained under 850°C (e.g., NK‐M_3_, NK‐M_4_). More interestingly, as illustrated in Figure 2d, those (002) peaks of NK‐M_3_ and NK‐M_4_ samples were located at 26.14° and 26.12°, which were negatively shifted from pristine 2θ location (≈26.4°), suggesting an average expanded *d*‐spacing (theoretically range from 0.337–0.340 nm) feature according to Bragg's law (2d sinθ = nλ).

Figure 2e demonstrates the results of Raman spectroscopy for the obtained carbon materials. The D band located ≈1350 cm^−1^ related to disordered structures or defects originates from sp^2^‐hybridization carbon, the G band located at ≈1580 cm^−1^ is associated with in‐plane vibration of sp^2^‐hybridization carbon, and the 2D band located at ≈2700 cm^−1^ manifests the stacking order of graphene layers, whereas the ratio (I_D_/I_G_) between the intensity of D band and G band is an indicator for graphitization degree. For instance, a lower I_D_/I_G_ ratio corresponds to a higher degree of graphitization [32]. The results clearly verified that the carbon product obtained under a stepwise CO_2_ electrolysis exhibited a better graphitization degree because of a relatively lower I_D_/I_G_ value, particularly for those obtained under 850°C (e.g., NK‐M_3_ and NK‐M_4_ samples, with ≈0.3). As a comparison, the I_D_/I_G_ value of the carbon samples without alkali metal activation (i.e., NK) reached 1.09, suggesting much more defects and undeveloped graphitic crystallinity. Moreover, the clearly visible 2D peaks further suggested the presence of multilayered graphene in the carbon materials after alkali metal activation treatment, while that delivered weak intensity for the carbon samples without treatment. Meanwhile, our previous investigation has verified that the catalytic graphitization effect of Ni electrode on carbon materials was prominent only under low current densities and over short reaction durations during CO_2_ electrolysis [23]. As depicted in XRD pattern (Figure 2c), no evident graphite features of the obtained carbon product (i.e., NK) were observed merely after CO_2_ electrolysis (Step 1), thus the catalytic graphitization effect of Ni on carbon materials should be negligible. Given the consideration above, it is worth‐noting that alkali metal activation treatment (Step 2), particularly conducted at an elevated temperature (e.g., 850°C), is beneficial to the graphitization of carbon products.

**FIGURE 3:**
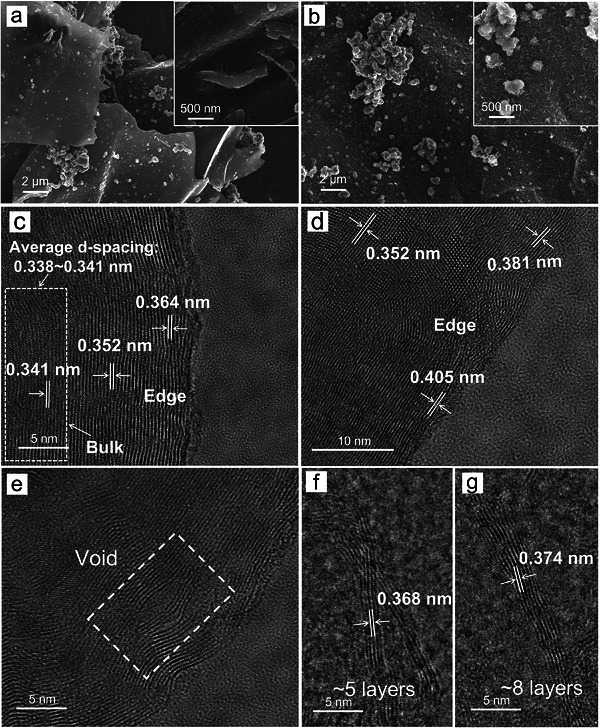
Electron microscopy of carbon materials obtained under different conditions. SEM images of NK (a) and NK‐M_4_ (b). (c–g) TEM images of NK‐M_4_.

To elucidate the possible origin of microstructures, SEM images show more details of morphologies of the obtained carbon materials. As shown in Figure 3a, the pristine morphology of as‐formed carbon sample (i.e., NK) derived from CO_2_ during Step 1 was primarily consisted of sheet‐like carbon flakes. Noting that a pre‐oxidized FeNi_36_ alloy plate with protective oxide scales was used as the anode (Figure S7a), the impact of transition metal originating from anode on pristine carbon morphology can be neglected (see carbon fibers using a raw FeNi_36_ alloy without pre‐oxidation treatment in Figure S7b, c). The carbon materials after stepwise CO_2_ electrolysis (Figure S8) exhibited similar sheet‐like morphology to NK at a macro‐scale perspective, however, their surface morphology differed significantly at a micro‐scale viewpoint. Particularly, the surfaces displayed wrinkle appearance with honeycomb‐like structures (Figure 3b and Figure S8c), which are similar to the typical features of graphite [33], indicating a higher degree of graphitization.

To gain more insights on the crystallinity and their atomic arrangement, TEM images show more straightforward evidences. As shown in Figure 3c,d, well‐defined lattice fringes belonging to graphite are observed, confirming a relatively high degree of graphitization. Compared to the regular interlayer *d*‐spacing (0.334 nm) of graphite, more interestingly, the carbon materials after stepwise CO_2_ electrolysis (e.g., NK‐M_4_) exhibited a noticeable increase in lattice *d*‐spacing (0.35–0.40 nm). As illustrated in Figure 3c, the interlayer spacing of graphite in the bulk region of the carbon material predominantly aligns within 0.338 to 0.341 nm, consistent with the theoretical value (from ≈0.337 to ≈0.340 nm) calculated using Bragg's law based on the peak shift in the XRD pattern. Moreover, it seems that the distribution of enlarged *d*‐spacing follows a location principle: the closer to the edge of carbon bulks the interlayer is, the more expanded *d*‐spacing of interlayer is, the interlayer spacing at the edge region of the carbon material can even reach 0.405 nm (Figure 3d). This phenomenon may be attributed to the contrast in the amount of in situ as‐formed metallic Na at different locations. Furthermore, as shown in Figure 3e, some voids originating from the expansion of interlayer *d*‐spacing were found, which should be attributed to the in situ exfoliation effect triggered by the as‐formed alkali metals. Owing to this unique process, few‐layered graphene consisting of 5–10 layers with expanded *d*‐spacing feature were also observed (Figure 3f, g). These observations are well aligned with the results of XRD and Raman spectra.

Considering that a lower operating temperature of CO_2_RR (Step 1) might benefit product selectivity toward carbon based on thermodynamic evaluation (Figure 1a), molten Na_2_CO_3_‐NaCl was selected as another electrolyte candidate due to its lower melting point (T_mp_ = 635°C), where the electrolysis product obtained with and without alkali metal activation (same condition to NK‐M_4_) was denoted as NN‐M_4_ and NN, respectively, gaining more insights on the technic adaptability of stepwise CO_2_ electrolysis. Owing to the lower melting point, CO_2_ electrolysis can also be performed at 650°C in molten NaCl‐Na_2_CO_3_, which allows for a higher CO_2_ content (e.g., 20 vol%) that favors carbon production. The results demonstrated a promoted current efficiency of up to ≈80% under 20 vol% CO_2_ atmosphere, which is consistent with our hypothesis, suggesting a promising application prospect for high‐efficiency conversion of CO_2_. Similar to the results in molten Na_2_CO_3_‐K_2_CO_3_, EDS analysis also indicated a decrease in oxygen content for the carbon sample with alkali metal activation treatment (i.e., NN‐M_4_) compared to the sample without treatment (i.e., NN) in molten Na_2_CO_3_‐NaCl. XRD patterns (Figure S9a) also revealed shifted diffraction peaks for NN‐M_4_, suggesting graphite with expanded *d*‐spacing feature, which is in good agreement with the observations in Raman spectroscopy (Figure S9b), SEM (Figure S9d) and TEM (Figure S9e–g). Given the consideration above, this proposed stepwise CO_2_ electrolysis strategy demonstrates an excellent adaptability. In addition, our pre‐experimental findings confirmed that reducing the electrolytic charge—specifically the current density and electrolysis duration—of Step 2 can also yield interlayer‐expanded graphite. Consequently, further optimization of total energy consumption can be achieved by refining the energy usage in Step 2.

### 2.3 Electrosynthesis Mechanism of Interlayer‐Expanded Graphite Derived from CO_2_

To gain more evidence on the significant role of alkali metal toward enlarged interlayers, a graphite rod was deliberately used as a cathode and electrolysis parameters that are identical with NK‐M_4_ and NN‐M_4_ during alkali metal activation (Step 2) were applied. As a result (Figure S10a), the pristine diffraction peak related to plane (002) in XRD pattern accordingly shifted after electrolysis, showing straightforward evidence, which is consistent with the observation in TEM image (Figure S10b, c). Moreover, as can be seen in Figure S10a, the negative shift of diffraction peak of graphite obtained after alkali metal activation treatment for 2 h was more evident than that for 0.5 h, suggesting that increasing electrolysis duration of alkali metal activation process can lead to graphite product with larger *d*‐spacing interlayers.

**FIGURE 4:**
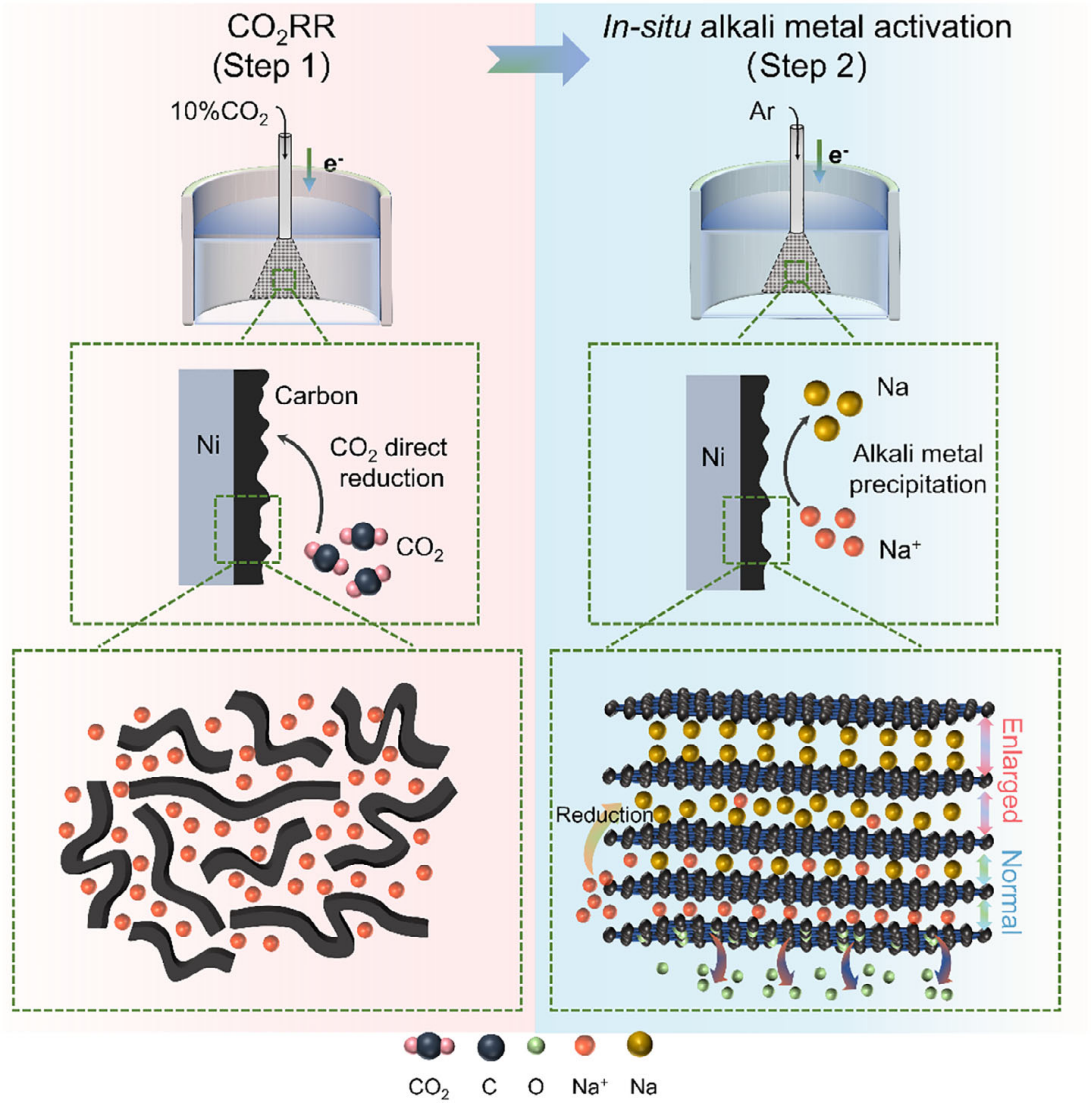
Mechanism for stepwise electrosynthesis of expanded *d*‐spacing graphite derived from CO_2_.

As illustrated in Figure 4, based on above‐mentioned findings, the mechanism for electrochemically synthesizing expanded *d*‐spacing graphite derived from CO_2_ can be depicted as follows: at Step 1, a low‐content CO_2_ (e.g., 10%) is electrochemically reduced at three‐phase (gas/liquid/solid) interface of a high‐temperature gas diffusion electrode (e.g., Ni foam electrode), generating amorphous carbon flakes with a relatively high oxygen content, where alkali metal ions (e.g., Na^+^) will be pre‐embedded into the as‐formed carbon interlayers by a more convenient way. At Step 2, when a strong electrode polarization that allows electroreduction of alkali metal ions to metallic alkali metal is applied, the pre‐embedded Na^+^ (diameter 0.204 nm) among carbon interlayers will transform into metallic Na (diameter 0.372 nm). Owing to the volumetric expansion, a strong stress will be released to squeeze surrounding interlayers, generating exfoliation effect [34]. The volume‐expansion‐driven exfoliation effect that enlarges the interlayer spacing of graphite is dependent on the atomic species of the applied expanding agent, which is associated with the ability contrast to overcome the van der Waals forces between the adjacent interlayers [35, 36]. It should be noted that an electro‐deoxidization process that help improve graphitization degree by removing oxygen will also occur during this period, so that the stress originating from metallic Na expansion can overcome van der Waals force between adjacent basal planes, leading to the enlarged *d*‐spacing of interlayer. Moreover, a shear force between liquid metal (liquid Na) and graphite interlayer also help exfoliation of graphite interlayers [37].

### 2.4 Electrochemical Performance as LIBs Anodes

**FIGURE 5:**
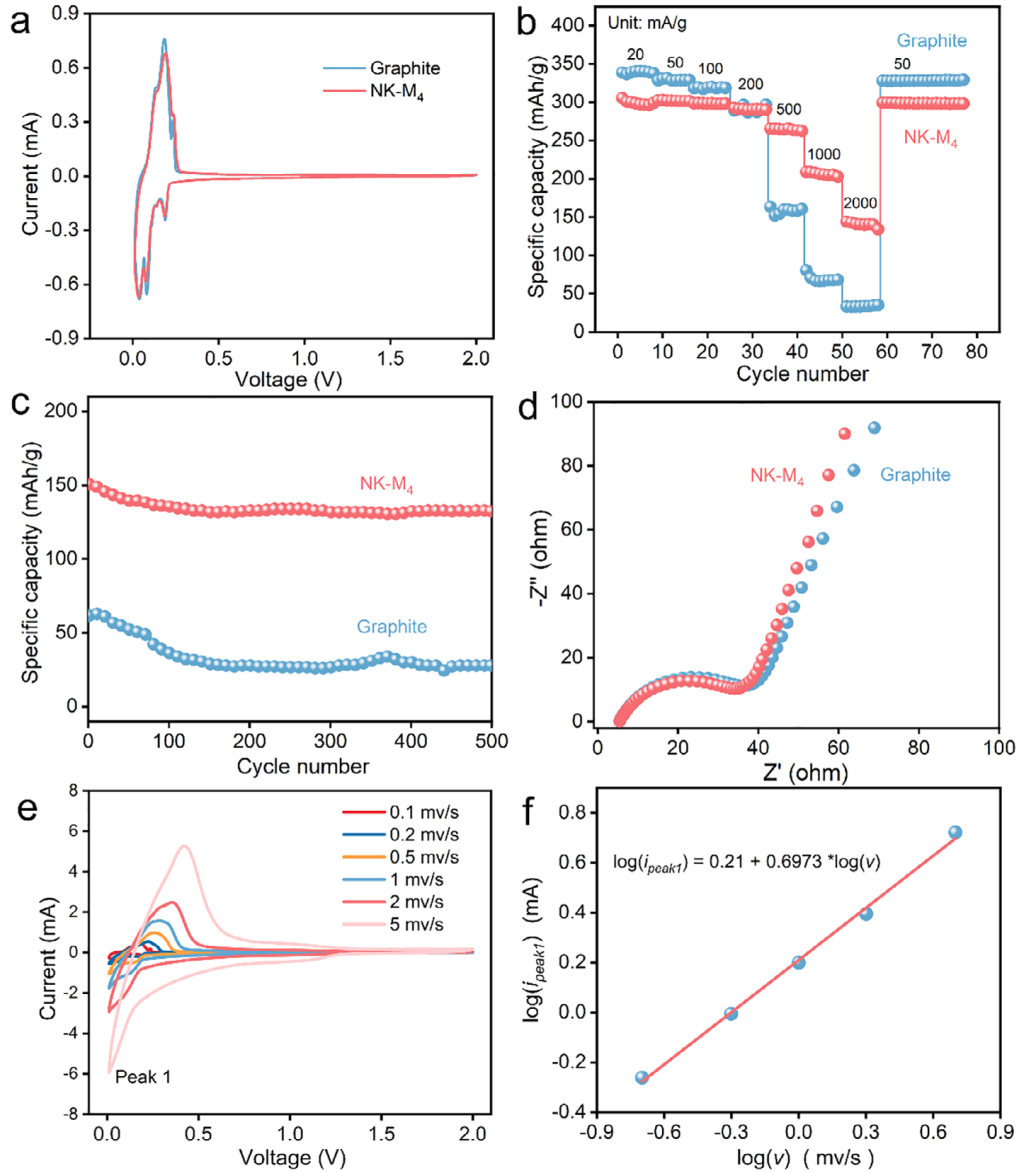
Lithium storage and electrochemical performance of NK‐M_4_ as a LIB anode in comparison with commercial graphite. (a) Cyclic voltammetry. Scan rate: 0.1 mV/s. (b) Rate capability. (c) Cycling performance at 2000 mA/g. (d) Nyquist plots of as‐prepared cells by electrochemical impedance spectroscopy. (e) Cyclic voltammetry profiles at various scan rates. (f) Linear correlations between peak current (log *i*) and scan rate (log *v*).

The electrochemical property of expanded *d*‐spacing graphite (NK‐M_4_) was examined by using a CR2032 coin type cell in a voltage range of 0.01–2.0 V. First, redox behaviors of the as‐prepared sample were evaluated by cyclic voltammetry (CV), as shown in Figure 5a. The NK‐M_4_ showed the similar electrochemical behavior with commercial graphite in the CV profile, with a series cathodic peaks, which are attributed to adsorption/intercalation of Li^+^ species into corresponding interlayers.

To demonstrate the effect of expanded interlayer spacing, we evaluated rate capability and corresponding long‐term galvanostatic cycling performances of NK‐M_4_ at a high rate. As shown in Figure 5b, c, by virtue of the expanded interlayer spacing, NK‐M_4_ sample retained excellent lithium storage properties at high current densities, reaching 152 mAh/g at 2000 mA/g after 500 cycles. Interestingly, conventional graphite anodes showed a very low capacity (≈30 mA h/g) at a current density of 2000 mA/g due to their narrow interlayer spacing. However, the capacity of NK‐M_4_ at low‐rate conditions was slightly lower than that of commercial graphite (Figure 5b), which can be attributed to the differences in the degree of graphitization. Specifically, the graphitization degree of NK‐M_4_ was likely not as perfect as that of commercial graphite. Enhancing the graphitization degree of NK‐M_4_ through an electro‐deoxidization process during electrolysis could improve its low‐rate performance [33]. Additionally, future work will also investigate the potential trade‐off between rate capability and capacity. Despite the presence of some impurities (e.g., Na, K), it should be pointed out that the samples demonstrate excellent cycling performance with 91% capacity retention after 500 cycles. Considering that the impurity levels in the carbon materials were relatively low, thus their influence on the electrochemical performance should be negligible.

To reveal charge transport behavior, we performed electrochemical impedance spectroscopy (EIS) analyses for NK‐M_4_ to gain deeper insights for the impact of interlayer distance on Li^+^ diffusion behaviors. Figure 5d displays the Nyquist plots of brand‐new NK‐M_4_, which was consist of a single semicircle at the high frequency region, and a straight line at the low frequency region was observed, corresponding to the resistance of Li^+^ diffusion through electrolyte/electrode interfaces (R_CT_) for the desolvation of Li^+^ ion, and Li^+^ diffusion impedance inside the active materials, respectively. Owing to the enhanced *d*‐spacing interlayer, the charge‐transfer resistance (R_CT_) of NK‐M_4_ decreased compared to commercial graphite. Besides, the higher slope of the straight line at the low frequency region implies better diffusion behavior [38]. Except for commercial graphite, NK‐M_4_ sample also exhibited superior high‐rate LIB performance and initial Coulombic efficiency of 85% compared with other typical carbon products derived from CO_2_ by molten salt CO_2_ electrolysis (Figures S11 and S12).

The ion storage mechanism was elucidated by carrying out CV sweep analysis at different scan rates from 0.1 to 5 mV/s (Figure 5e). The charge storage mechanisms are typically classified into two reactions of capacitive‐controlled reaction and diffusion‐controlled reaction, and both can be described by a power law relationship along the scan rate [39]:

(6) $$i=av^{b}$$

where *i* is the measured current (peak 1 in Figure 5e), *v* is the scan rate, and a and b are adjustable parameters. The b value close to 0.5 implies a diffusion‐controlled process while that close to 1 refers a capacitive process. As a result, the b values for NK‐M_4_ reached ≈ 0.7 (Figure 5f), suggesting a lithium storage mechanism with mixed capacitance and diffusion control that the expanded layered structure contributes to facile ion intercalation reactions and offers numerous electroactive reaction sites accommodating Li^+^ diffusion besides a diffusion‐controlled process. This synergy between the capacitive‐controlled reaction and the diffusion‐controlled reaction results in a fast lithiation/delithiation process as well as a large ion storage capacity, particularly at high rates.

## 3 Conclusions

This work achieved electrosynthesis of CO_2_‐derived graphite with expanded *d*‐spacing interlayers enabled by a high‐temperature gas diffusion electrode via a stepwise electrolysis strategy, where dilute CO_2_ (10%–20%, close to practical flue gas) was directly electro‐reduced coupled by an sequential in situ alkali metal activation process. Thermodynamic analysis confirmed carbon materials were the dominant CO_2_RR products, of which crystallinity and *d*‐spacing were promoted with the evolution of alkali metals. The proposed stepwise CO_2_ electrolysis strategy is a prototype method demonstrating a wide adaptability in different molten electrolyte systems. The current efficiency for acquiring the target graphite can reach as high as over 80% under the optimized condition. Owing to the in situ exfoliation effect and shear effort induced by alkali metals, the graphite exhibited expanded *d*‐spacing interlayer of 0.338–0.405 nm, showing excellent performance for rapid lithium storage, 206 and 152 mAh/g at high current densities of 1000 and 2000 mA/g, respectively, with over 91% capacity retention after 500 cycles. This work not only deepens the fundamental understanding of the direct utilization of CO_2_, particularly under a dilute CO_2_ content, but also provides new insights for rational design of carbon anode materials toward next‐generation lithium‐ion battery industry at a lower carbon footprint level.

## 4 Methods

### 4.1 Materials

500 g of Na_2_CO_3_–K_2_CO_3_ (59:41 mol%, melting point T_mp_ ≈ 709°C) mixture was selected as an electrolyte candidate, which was placed in an alumina crucible and was heated to the target operating temperature (e.g., 750, 850°C) at 5°C/min in a vertical stainless‐steel furnace under an Ar atmosphere (a flow of 100 mL/min). A pre‐oxidized FeNi_36_ alloy sheet (1.5 cm × 1.5 cm) with protective oxide scale was used as an inert anode [40], and a home‐made high‐temperature gas diffusion electrode (HT‐GDE) was used as a cathode, which was made of a funnel‐shaped Ni foam centered with a hollow stainless‐steel tube (concurrently acted as current collector and gas delivery channel), see the schematic illustration in Figure S1. All the chemical agents were supplied by Sinopharm Chemical Reagent Co., Ltd., with a purity of ≥99.0%.

### 4.2 Thermodynamics and Electrochemical Measurements in Molten Electrolyzer

The data of Gibbs free energy for thermodynamic calculations were obtained using HSC Chemistry software. To investigate the electrochemical behaviors in the molten electrolyte system, a three‐electrode configuration was employed. Specifically, a Ni foam sheet (2.0 cm × 2.0 cm) and a graphite rod (contact area of 6.48 cm^2^) acted as a working electrode and a counter electrode, respectively. A well‐developed Ag_2_SO_4_/Ag reference electrode was used to indicate the relative potential of working electrode [29], of which potential was normalized to that of alkali metal evolution (vs. Na^+^/Na). Cyclic voltammetry (CV) measurements were carried out with an electrochemical workstation (CS310, CorrTest Instruments Co., Ltd., China).

### 4.3 Stepwise Electrolysis Process for Synthesis of CO_2_‐Derived Carbon

The proposed stepwise electrolysis strategy was classified into two sequential periods: CO_2_ electrolysis (Step 1) and alkali metal activation (Step 2). To be specific, at Step 1, CO_2_ electrolysis was carried out at 100 mA/cm^2^ for 1 h by galvanostatic electrolysis under a CO_2_‐containing atmosphere at 750°C. To achieve a continuous and stable CO_2_‐to‐carbon conversion, a well‐defined HT‐GDE was used, where 10% CO_2_ (balanced with Ar, 150 mL/min) was consistently introduced by a hollow stainless steel tube (acted as a current collector) centered over the top of funnel‐shaped Ni foam during CO_2_ electrolysis. After that, electrolysis was carried out by constant‐voltage electrolysis under a CO_2_‐free (i.e., Ar) atmosphere to achieve alkali metal evolution at Step 2. The electrolysis process was conducted using a DC power supply (Neware Electronics Ltd., China). The variations of operating temperature and cell voltage toward carbon product property were also investigated. To investigate the adaptability of the stepwise electrolysis toward target carbon product, the electrolysis was also carried out in molten Na_2_CO_3_‐NaCl (58:42 mol%, T_mp_ ≈ 635°C) as a comparison. After electrolysis, the cooled cathode along with the carbon deposits were immersed into deionized water to peel off the adhered carbon product and remove the frozen but water‐soluble electrolyte. Afterwards, the mixture was filtered to separate solid carbon, where the obtain carbon products were dried in an oven at 80°C for 12 h for further characterizations. The carbon products obtained in molten Na_2_CO_3_‐K_2_CO_3_ under different electrolysis conditions were denoted as NK‐M_x_ (see the abbreviation index details in Table S1).

### 4.4 Material Characterization

The crystal phase structure of the obtained carbon materials was determined by X‐ray diffraction (XRD; Rigaku Mini Flex 600, Cu Kα_1_ radiation). To investigate the surface morphology and graphitic lattice spacing, field emission scanning electron microscopy (FE‐SEM; Sigma 300) and transmission electron microscopy (TEM; JEOL JEM‐NEOARM, operated at 200 kV) were employed. The degree of graphitization was quantified using Raman spectroscopy (LabRAMHR evolution, 532 nm excitation wavelength). Elemental composition (C, O, and trace amounts of transition metals) was analyzed by energy‐dispersive X‐ray spectroscopy (EDS; FEI Quanta FEG 250).

### 4.5 Cell Assembly and Electrochemical Measurements for LIB

The electrochemical performance of carbon products as LIB anode materials was carried out by a half‐cell test, which was performed using CR2032 coin cells assembled in an Ar‐filled glove box. The working electrode was prepared by mixing the active material, polyvinylidene fluoride (PVDF) binder, and conductive carbon black in a weight ratio of 8:1:1, using N‐methyl‐2‐pyrrolidone (NMP) as the solvent. The loading mass of the active materials was approximately 1.2–1.5 mg/cm^2^. The coin cells consisted of lithium foil as a counter electrode, a polyethylene separator (Celgard 2400), and 1.0 M LiPF_6_ electrolyte in a mixture of ethylene carbonate (EC), diethyl carbonate (DEC), and ethyl methyl carbonate (EMC) (1:1:1 by volume). Galvanostatic charge–discharge tests were conducted at 25°C using a LAND2001CT system. Cyclic voltammetry (CV) and electrochemical impedance spectroscopy (EIS) were performed on an electrochemical workstation (CH Instruments, Shanghai Chenhua Co.) within a voltage window of 0.01–2.0 V (vs. Li^+^/Li). The EIS measurements were carried out with an AC amplitude of 5 mV over a frequency range from 100 kHz to 10 mHz.

## Author Contributions

**Hao Zha**: methodology, investigation, validation, formal analysis, writing – original draft, visualization. **Xinyu Li**: methodology, validation. **Dihua Wang**: validation, project administration, resources, supervision. **Huayi Yin**: methodology, validation, supervision, resources, formal analysis. **Xiaodan Zhang**: software, data curation, validation, methodology, investigation, visualization. **Yuxin Wu**: methodology, validation, formal analysis. **Bowen Deng**: conceptualization, methodology, formal analysis, supervision, funding acquisition, project administration, resources, writing – original draft, writing – review and editing, data curation, validation. **Xiaoyang Wang**: formal analysis, visualization. **Jiajun Li**: methodology, data curation, formal analysis.

## Conflicts of Interest

The authors declare no conflicts of interest.

## Supporting information




**Supporting File**: advs76734‐sup‐0001‐SuppMat.docx.

## Data Availability

The data that support the findings of this study are available from the corresponding author upon reasonable request.
