# Supplementary material for: In Situ Alkali Metal Exfoliation‐Coupled Dilute CO2 Electrolysis to Synthesize Interlayer‐Expanded Graphite for High‐Rate Lithium Storage
Source: Adv Sci (Weinh). 2026 Jul 23:e76734. Online ahead of print. doi: 10.1002/advs.76734 (PMC13393327; doi:10.1002/advs.76734)
Supplement: Supplementary file 1 — Supporting File: advs76734‐sup‐0001‐SuppMat.docx. [file ADVS-9999-e76734-s001.docx]

**Supplementary Information**

***In-Situ* Alkali Metal Exfoliation-Coupled Dilute CO_2_ Electrolysis to Synthesize Interlayer-Expanded Graphite for High-Rate Lithium Storage**

Hao Zha, Xiaodan Zhang, Xinyu Li, Jiajun Li, Yuxin Wu, Xiaoyang Wang, Huayi Yin, Dihua Wang, Bowen Deng^*^

School of Resource and Environmental Sciences, Wuhan University, Wuhan 430072, P. R. China.

Hubei International Scientific and Technological Cooperation Base of Sustainable Resource and Energy

^*^Email: bwdeng@whu.edu.cn

**Thermodynamic correlation between theoretical potential and CO_2_ partial pressure**

Thermodynamic calculation formula for the influence of gas partial pressure on the cathode potential of direct CO_2_ reduction:

$E_{{CO}_{2}/C}=E_{{CO}_{2}/C}^{\theta}+ \frac{2.303\cdot RT}{nF}\lg\frac{P({CO}_{2})}{p^{0}}$ (S1)

$E_{{CO}_{2}/CO}=E_{{CO}_{2}/CO}^{\theta}+ \frac{2\cdot2.303\cdot RT}{nF}\lg\frac{P({CO}_{2})}{p^{0}}$ (S2)

$E^{\theta}$ for cathodic reaction compared to the standard electrode potential of Na^+^/ Na; *R* is the gas constant, 8.314 J/(K·mol); *T* represents the reaction temperature, K; n is the number of transferred electrons; *F* is Faraday's constant, 96485 C/mol; *P^0^* represents 1 atm. To simplify the calculation, all variables except the partial pressure (P(CO_2_)) of CO_2_ are set to the standard state.

**The calculation of current efficiency**

$\eta=\frac{m}{M}\cdot\frac{nF}{Q}\cdot100\%$ （S3）

where *m* (g) and M (g/mol) represent the actual carbon product mass and its molar mass (12 g/mol), respectively. *n* refers to the electron transfer number (n=4); F (C/mol) denotes to Faraday constant (96485 C/mol); Q (C) stands for the actual charge passed during the electrolysis.

**Synthesis of carbon products by conventional molten salt CO_2_ electrolysis**

To highlight the unique structures and lithium-ion battery (LIB) anode performance of the target carbon product—specifically, expanded *d*-spacing graphite produced via direct CO_2_ electrolysis enabled by a high-temperature gas diffusion electrode (HT-GDE)—we also synthesized carbon products (denoted as LNK) in a molten Li_2_CO_3_-Na_2_CO_3_-K_2_CO_3_ (43.5:31.5:25 mol%, T_mp_ ≈ 397 ^o^C) mixture, which is one of the most typical molten salt electrolytes for indirect CO_2_-to-carbon conversion. A Ni planar sheet (3 cm × 3 cm) and NiFe36 alloy sheet acted as cathode and anode, respectively. The electrolysis for synthesizing carbon products was conducted at 100 mA/cm² and 750 °C, using electrolysis parameters identical to those of the NK sample (i.e., CO_2_RR at Step 1 in molten Na_2_CO_3_-K_2_CO_3_). It is important to note that no alkali metal activation process was applied. The comparison of LIB anode performance can be found in Figure S11 and 12.


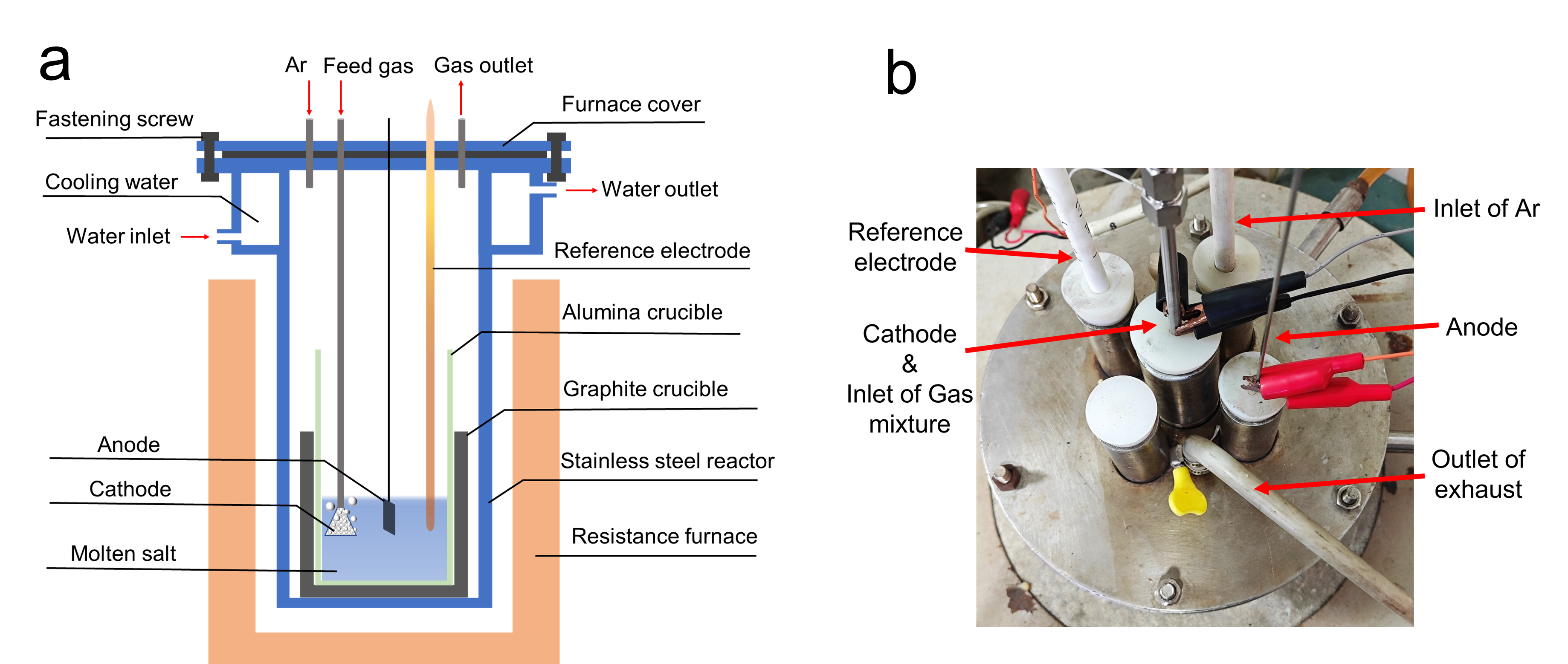


**Figure S1**. (a) Cross-section view of electrolysis reactor. (b) Optical images of top view of the reactor.


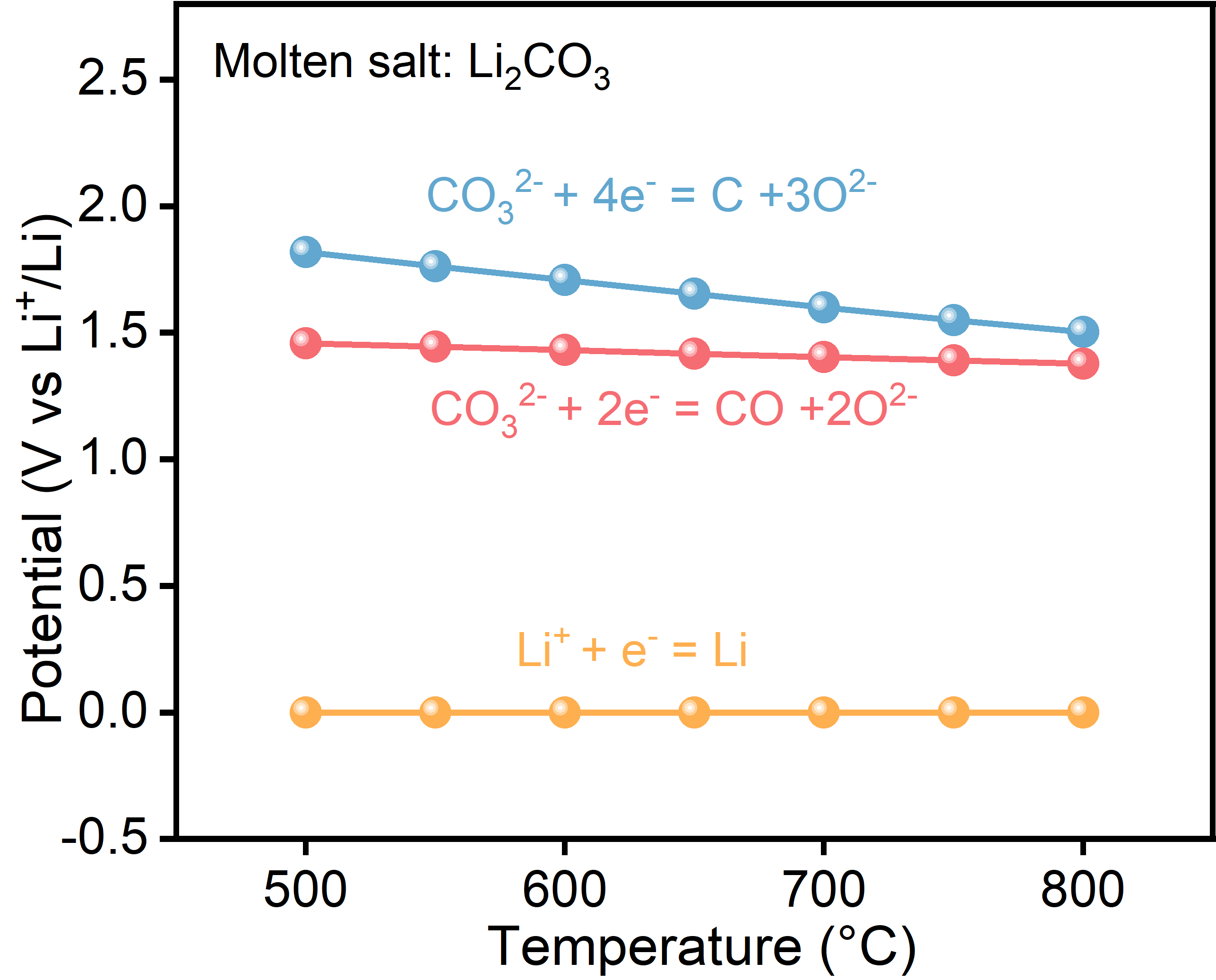


**Figure S2.** Theoretical potentials of possible cathodic reactions in molten Li_2_CO_3_.


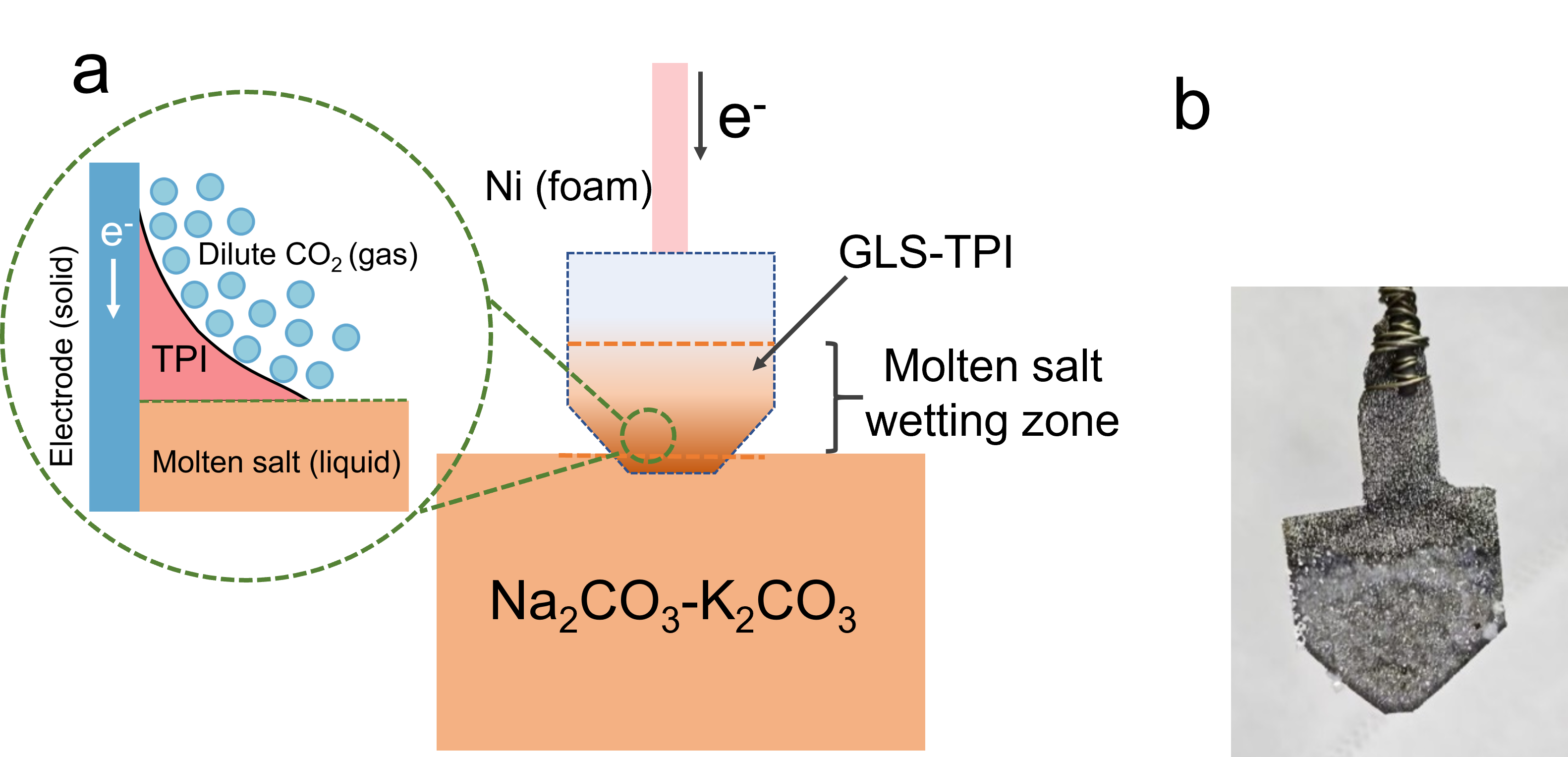


**Figure S3.** Schematic illustration of three-phase interface (TPI) of working electrode (a) and corresponding optical image (b).


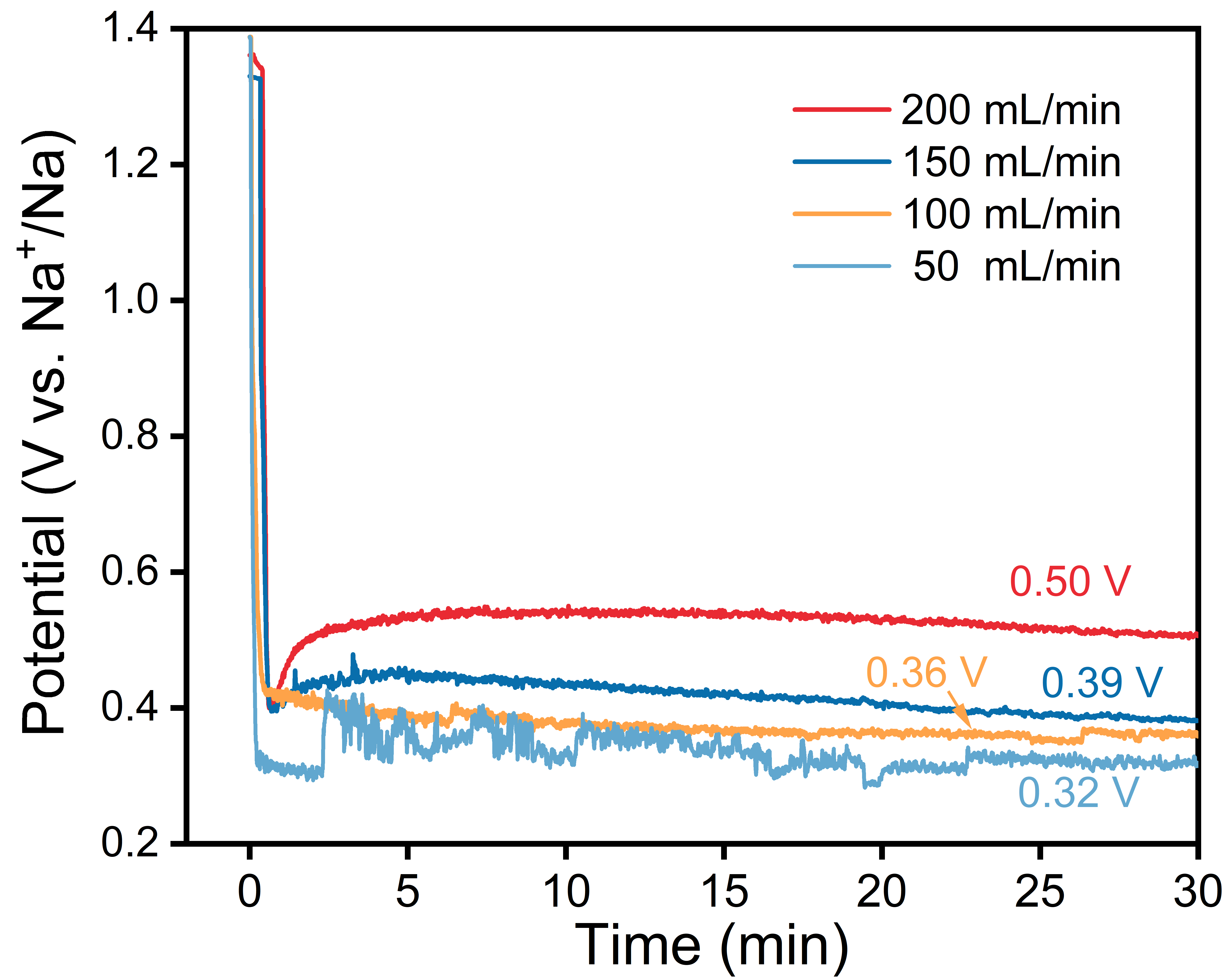


**Figure S4.** The influence of gas flow rate on cathodic potential.


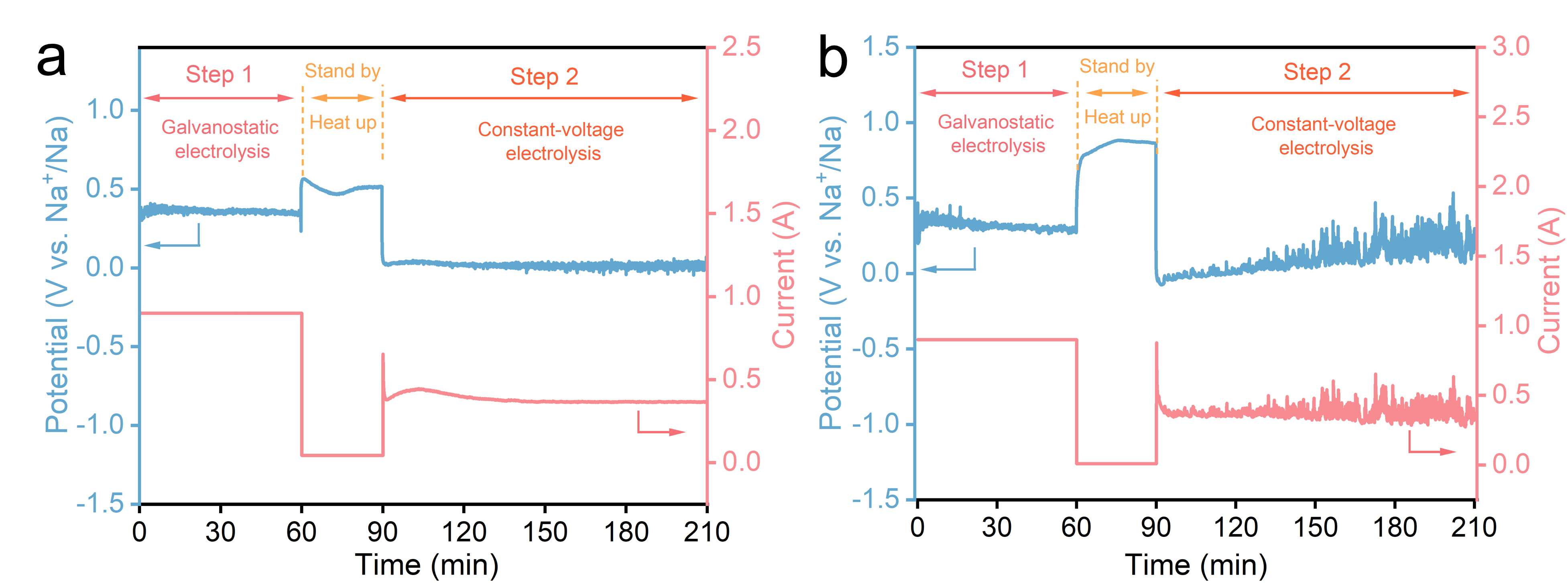


**Figure S5.** The variations of potential and current during stepwise CO_2_ electrolysis under different conditions. (a) NK-M_3_. (b) NK-M_4_.


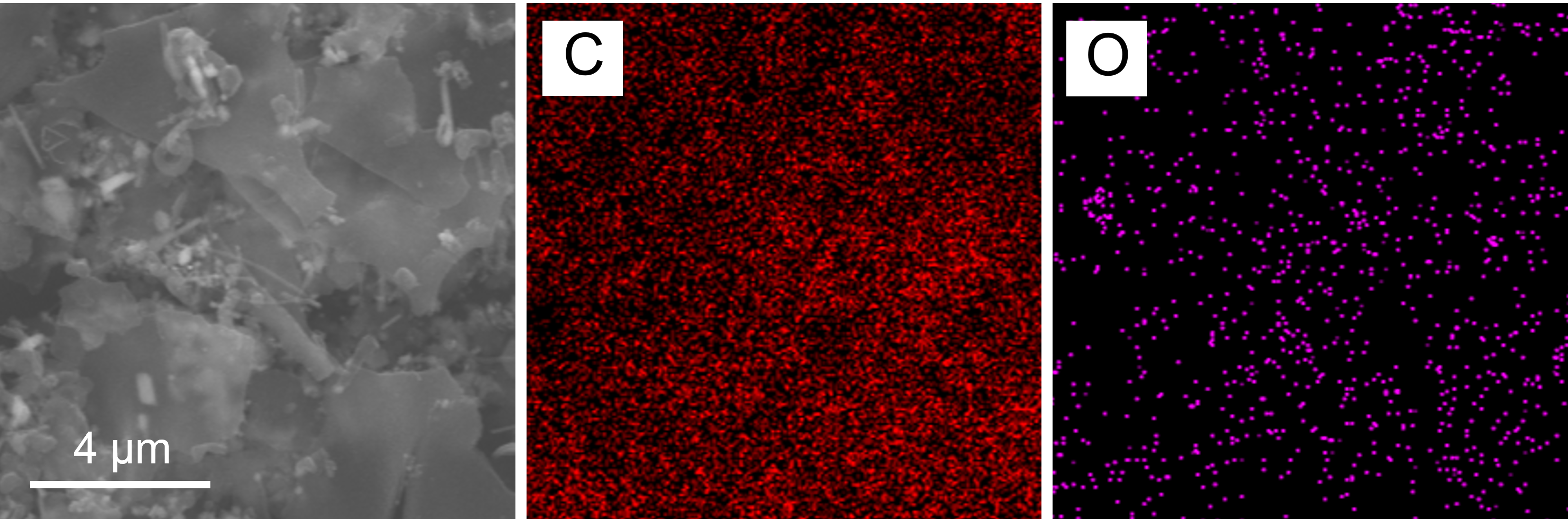


**Figure S6**. Mapping images of NK.


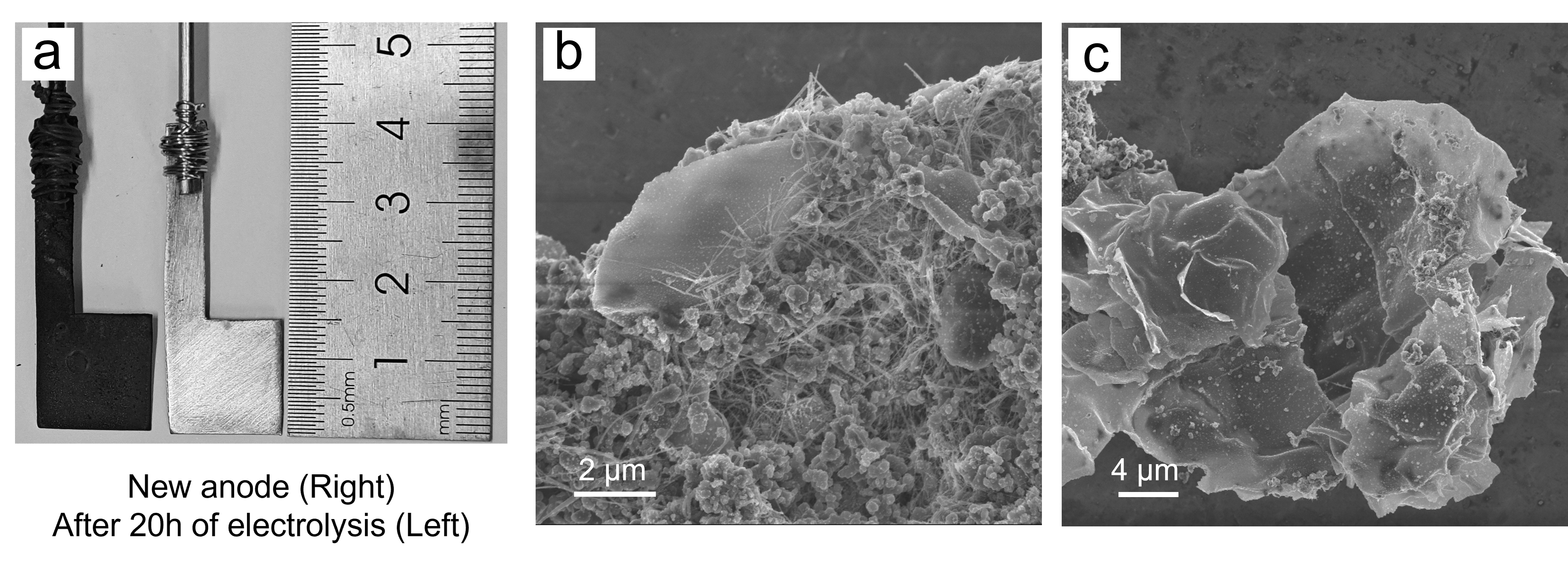


**Figure S7.** (a) Optical image of the FeNi_36_ anode. (b,c) SEM images of carbon products using a FeNi_36_ anode without pre-oxidation treatment.


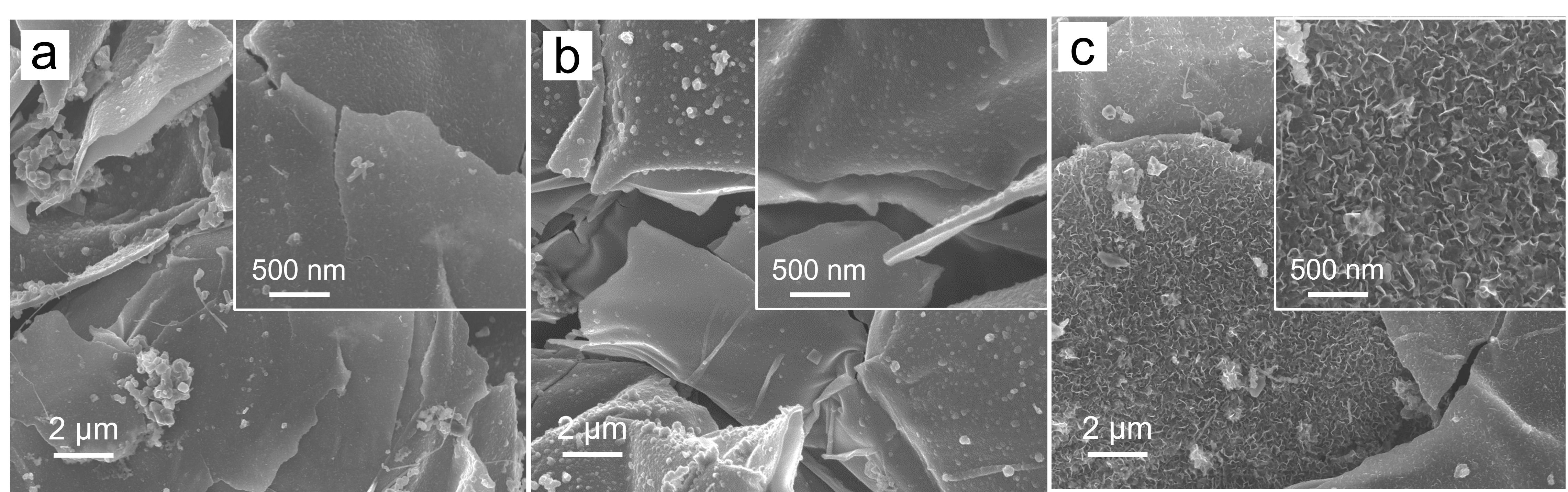


**Figure S8.** SEM images of NK-M_1_(a), NK-M_2_(b) and NK-M_3_(c).


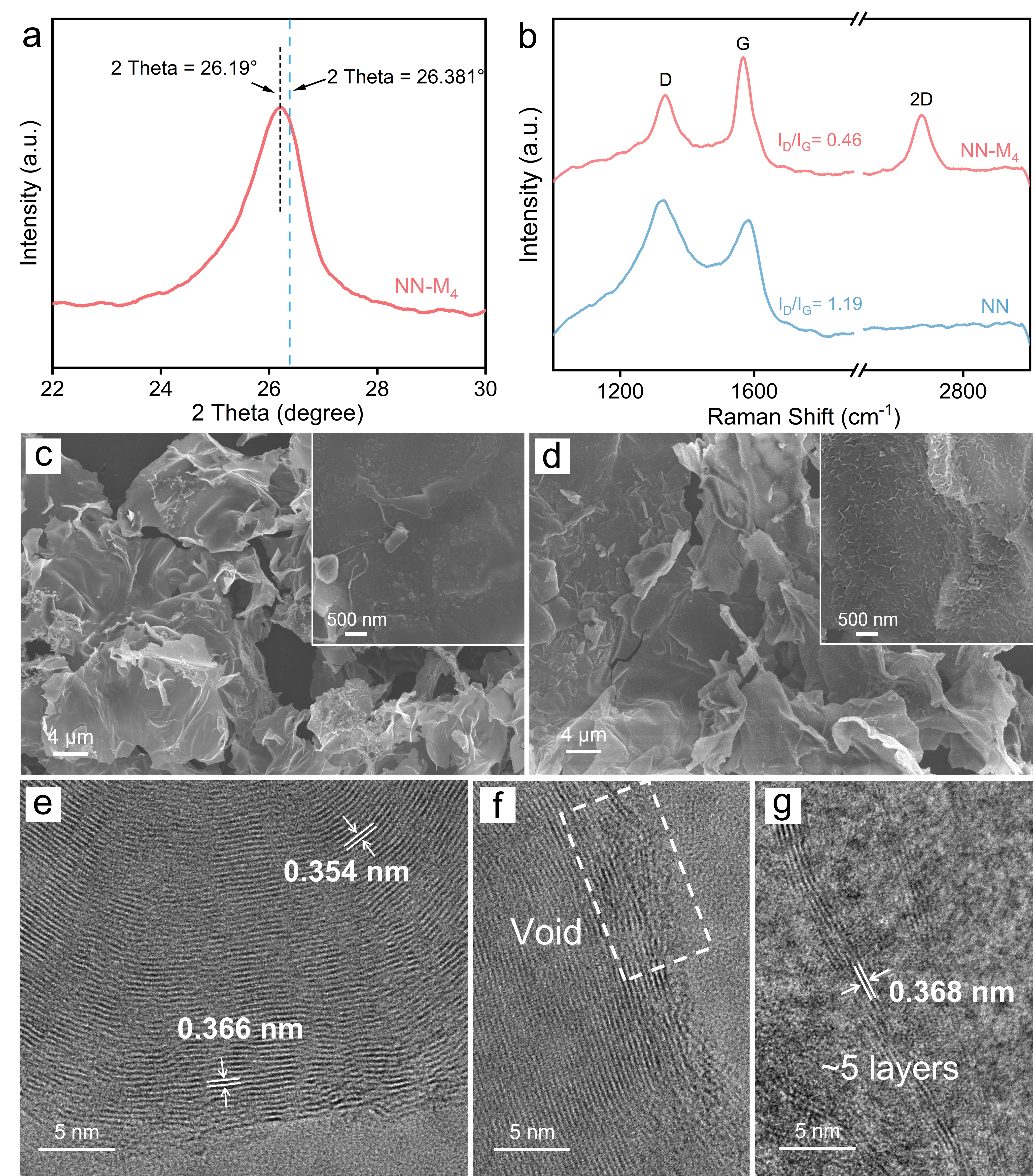


**Figure S9.**  XRD pattern (a) and Raman spectra (b) of different carbon materials. SEM images of NN (c) and NN-M_4_ (d); (e-g) TEM images of the NN-M_4_.


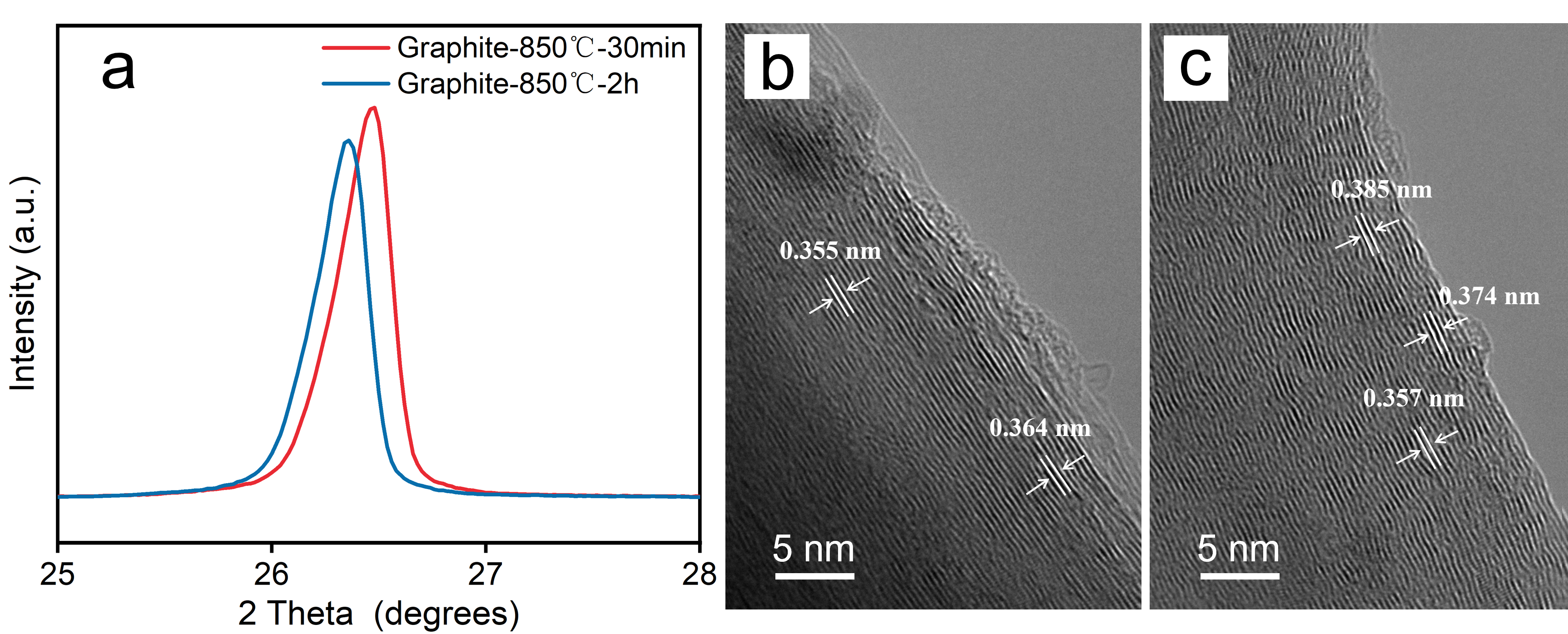


**Figure S10.** XRD patterns (a) and TEM images of obtained graphite after alkali metal activation for 30min (b) and 2h (c), respectively.


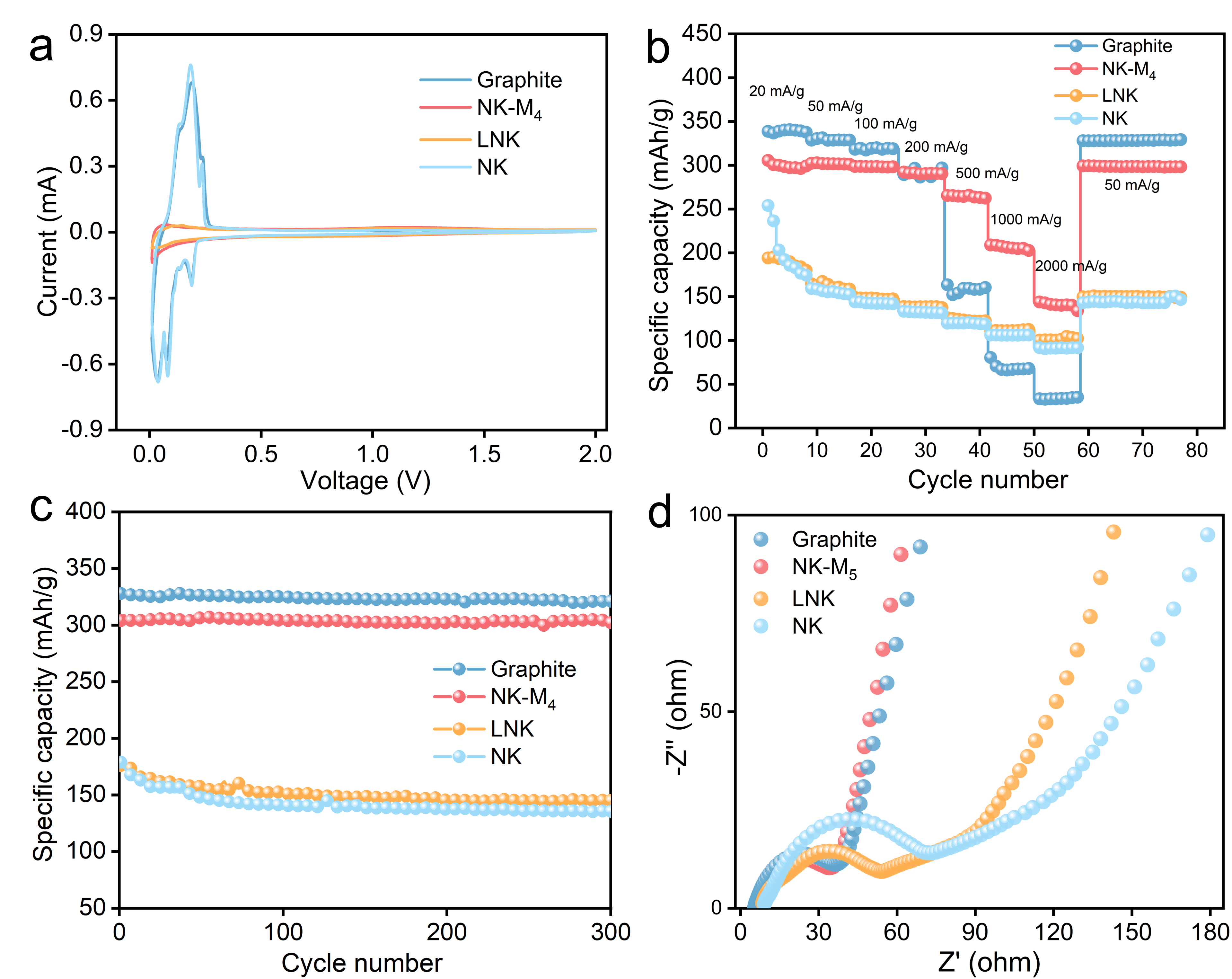


**Figure S11.** Lithium storage and electrochemical performance of graphite, NK-M_4_, LNK and NK as a LIB anode. (a) Cyclic voltammetry. Scan rate: 0.1 mV/s. (b) Rate capability. (c) Cycling performance at 100 mA/g. (d) Nyquist plots of as-prepared cells by electrochemical impedance spetroscopy.


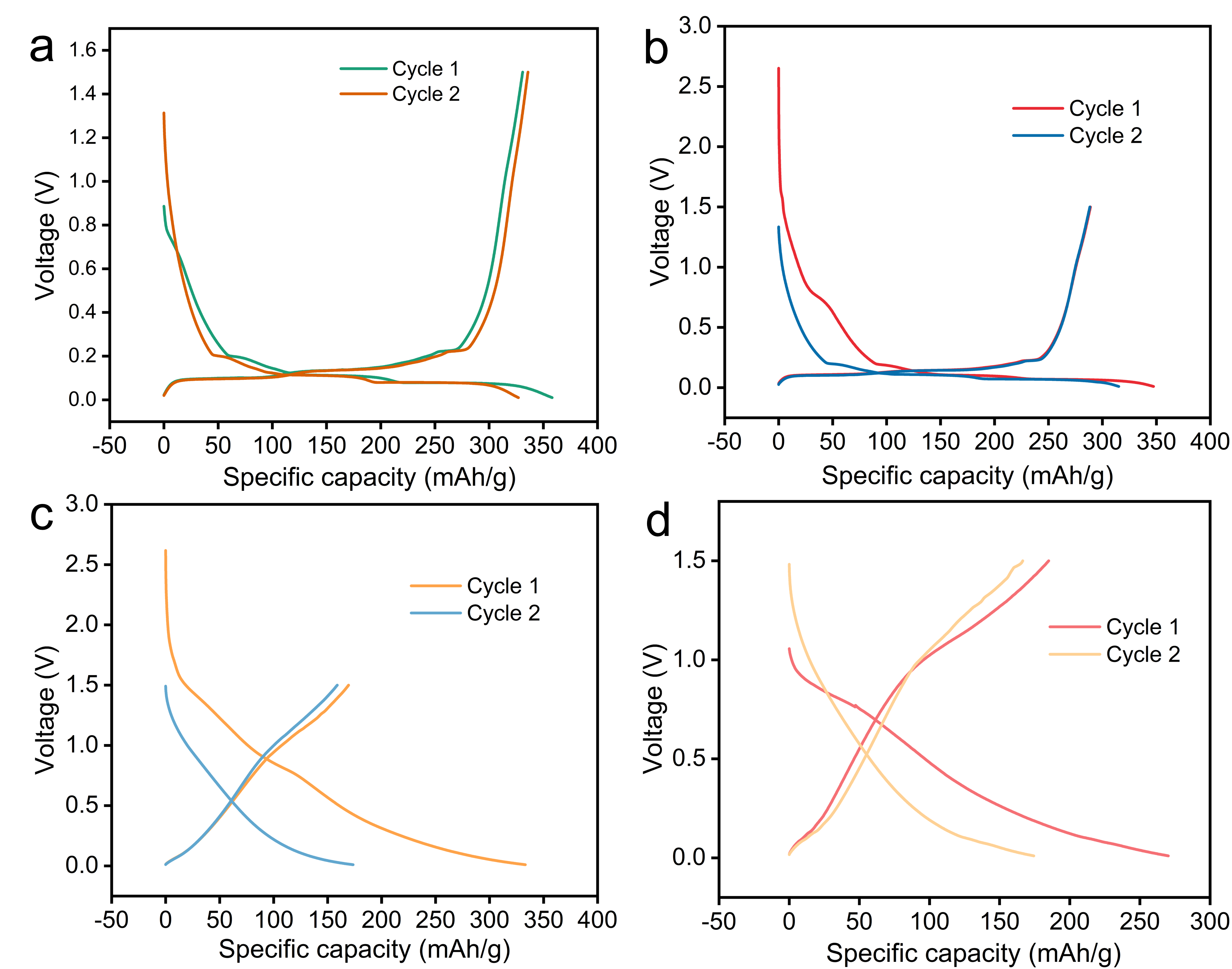


**Figure S12.** The charge-discharge curves of 1^st^ and 2^nd^ cycles at a current density of 100mA/g: Graphite(a), NK-M_4_(b), LNK(c), NK(d).

**Table S1.** The abbreviation of electrolysis parameters for carbon products obtained under different conditions using a high-temperature gas diffusion electrode.

| Electrolyte | **Step 1** CO_2_RR | **Step 2**  *In-situ* alkali metal activation | Abbreviation index |
| --- | --- | --- | --- |
| Na_2_CO_3_-K_2_CO_3_  (59:41 mo%) | 750 ^o^C, 10%-CO_2_  100 mA/cm^2^, 1 h | — | NK |
|  |  | 750 ^o^C, Ar, 2.4 V, 2 h | NK–M_1_ |
|  |  | 750 ^o^C, Ar, 2.4 V, 6 h | NK–M_2_ |
|  |  | 850 ^o^C, Ar, 2.2 V, 2 h | NK–M_3_ |
|  |  | 850 ^o^C, Ar, 2.4 V, 2 h | NK–M_4_ |
| NaCl-Na_2_CO_3_  (58:42 mol%) | 650 ^o^C, 20%-CO_2_  100 mA/cm^2^, 1 h | — | NN |
|  |  | 850 ^o^C, Ar, 2.4 V, 2 h | NN–M_4_ |

**Table S2.** Elemental composition of carbon materials using HT-GDE under different electrolysis conditions.

|  | At. % | | | | | | |
| --- | --- | --- | --- | --- | --- | --- | --- |
|  | C | O | Fe | Ni | Cr | Na | K |
| NK | 83.61 | 14.82 | 0.62 | 0.39 | 0.36 | NAN | NAN |
| NK-M_1_ | 86.58 | 12.70 | 0.47 | 0.19 | 0.06 | NAN | NAN |
| NK-M_2_ | 86.09 | 13.16 | 0.15 | 0.41 | 0.18 | NAN | NAN |
| NK-M_3_ | 91.93 | 6.80 | 0.47 | 0.26 | NAN | 0.24 | 0.31 |
| NK-M_4_ | 91.40 | 6.43 | 0.37 | 0.69 | 0.47 | 0.38 | 0.26 |
